# Drivers and fitness consequences of dispersive migration in a pelagic seabird

**DOI:** 10.1093/beheco/arw013

**Published:** 2016-02-17

**Authors:** Annette L. Fayet, Robin Freeman, Akiko Shoji, Dave Boyle, Holly L. Kirk, Ben J. Dean, Chris M. Perrins, Tim Guilford

**Affiliations:** 1 ^a^ Oxford Navigation Group, Department of Zoology, University of Oxford, South Parks Road, Oxford OX1 3PS, UK,; 2 ^b^ Institute of Zoology, Zoological Society of London, Outer Circle, Regents Park, London NW1 4RY, UK, and; 3 ^c^ Edward Grey Institute, Department of Zoology, University of Oxford, South Parks Road, Oxford OX1 3PS, UK

**Keywords:** Atlantic puffin, dispersion, fitness, geolocation, migration, route fidelity.

## Abstract

Animals can be flexible in their migration strategies, using several wintering sites or a variety of routes. The mechanisms promoting the development of these migratory patterns and their potential fitness consequences are poorly understood. Here, we address these questions by tracking the dispersive migration of a pelagic seabird, the Atlantic puffin *Fratercula arctica*, using over 100 complete migration tracks collected over 7 years, including repeated tracks of individuals for up to 6 consecutive years. Because puffins have high flight costs, dispersion may generate important variation in costs of migration. We investigate differences in activity budgets and energy expenditure between different strategies. We find that puffins visit a range of overwintering destinations, resulting in a diversity of migratory routes differing in energy expenditures; however, they show interindividual similarity in the timings and location of major movements. We consider 3 hypothetical mechanisms that could generate this pattern: 1) random dispersion; 2) sex segregation; and 3) intraspecific competition or differences in individual quality. First, we dismiss random dispersion because individuals show strong route fidelity between years. Second, we find that sex differences contribute to, but do not account fully for, the migratory variation observed. Third, we find significant differences in breeding success between overwintering destinations, which, together with differences in foraging levels between routes, suggest that birds of different quality may visit different destinations. Taken together, our results show that dispersive migration is a complex phenomenon that can be driven by multiple factors simultaneously and can shape a population’s fitness landscape.

## Lay Summary

Sex segregation, competition and differences in individual quality may drive dispersive migration in birds and affect their fitness. Atlantic puffins tracked for up to 6 years followed remarkably different migration routes, but individuals followed the same route every year. Although random dispersion and sex segregation could not explain the patterns observed, birds visiting the Mediterranean Sea foraged more and had a higher breeding success than birds remaining locally or visiting the Atlantic Ocean.

## INTRODUCTION

Typical long-distance migrant species move annually between a breeding ground and a single broad area where all individuals spend the winter (Dingle 1980; Newton 2008). However, migratory patterns can be more complex, with animals following different routes to the same wintering ground (Brower 1996; Papi et al. 2000; Hake et al. 2003) or wintering in different areas (McConnell and Fedak 1996; Boustany et al. 2002; Dias et al. 2011; McFarlane Tranquilla et al. 2014). This variation in individual migratory destinations and routes is exemplified in dispersive migrants, whose migration can occur in any direction from the breeding site but still involves a return journey (Newton 2008). Dispersive migration raises fundamental questions about how long-distance movements are controlled, and how they affect fitness and breeding ecology. Some routes may be more dangerous, energetically demanding or longer to follow, and lead to later breeding (Alerstam and Lindström 1990), and wintering grounds may differ in productivity. Such consequences have been scarcely studied (Sergio et al. 2014; Weimerskirch et al. 2015) and remain poorly understood.

Migrants with a population-wide single migratory direction are thought to inherit at least the direction and duration of their migration route genetically (Perdeck 1958; Helbig 1991; Berthold et al. 1992; Berthold 1996) or to learn it by following family members or other conspecifics (Chernetsov et al. 2004; Harrison et al. 2010; Palacin et al. 2011). Dispersive migration does not lend itself to control by either of these mechanisms (Guilford et al. 2011). Therefore, it is unknown what controls the directional decisions of migrants when these are highly variable within a single population. Several (not necessarily mutually exclusive) mechanisms could lead to dispersive migration. First, random dispersion from the breeding site, whereby each individual follows a random direction each year, could generate individual variability in migratory directions and lead to random differences among and within individuals, across years. There is no strong evidence of this to date documented in any species. Random dispersion may be a risky and less profitable strategy unless areas visited during the nonbreeding season have unpredictable resource availability or plentiful homogenous resources without major barriers or dangers. Comparing individuals’ migratory routes over multiple years can help determine whether they follow random directions each year, but studies so far have provided mixed results. Some species show a degree of fidelity to their route (Hunter et al. 2003; Sakuragi et al. 2004; Shiu et al. 2006; Broderick et al. 2007; Yamamoto et al. 2010), whereas others show high variability (Berthold et al. 2004; Alerstam et al. 2006; Dias et al. 2013).

A second potential driver of dispersive migration is sex segregation, which might occur if males and females differ in foraging niche or energy requirements (Selander 1966; Cristol et al. 1999) or as a result of intraspecific competition (Marra and Holmes 2001). Such segregation has mostly been observed in sexually dimorphic species (Brown et al. 1995; Stewart 1997; Phillips et al. 2011), but not always (Guilford et al. 2012; Müller et al. 2014). Competition could also act regardless of sex. Lesser-quality individuals may be forced to migrate further if local resources cannot sustain the whole population in winter (Gauthreaux 1982; Gunnarsson et al. 2005); conversely, only high-quality individuals may be able to travel to distant productive areas (Blake et al. 2013). The latter is not intraspecific competition per se but would result in a similar pattern. In either case, we expect there to be fitness consequences of variation in migration routes and distances (Klaassen 2003; Alves et al. 2013). Other potential drivers of dispersive migration, not specifically addressed here, include age-related differences (Jonsson et al. 1990; Cristol et al. 1999; Thiebot et al. 2011; Riotte-Lambert and Weimerskirch 2013), exploration in the early life followed by gradual refinement of the migratory route (Guilford et al. 2011), or individual specialization or variation (Bearhop et al. 2006; Phillips et al. 2011).

The current study aims to test the role of random dispersion, sex segregation, and intraspecific competition as potential drivers of dispersive migration in a pelagic seabird, the Atlantic puffin *Fratercula arctica*. Puffins are small North Atlantic seabirds that exhibit dispersive migration (Guilford et al. 2011; Jessopp et al. 2013), although this varies between colonies (Harris et al. 2010). The migration strategies of seabirds, although less well understood than those of terrestrial species, seem to show large variation in flexibility between species, making them good models to study flexibility in migratory strategies (Croxall et al. 2005; Phillips et al. 2005; Shaffer et al. 2006; Gonzales-Solis et al. 2007; Guilford et al. 2009). Here, we track the migration of over 100 complete migrations of puffins using miniature geolocators over 8 years. First, we investigate the role of random dispersion (or semirandom, as some directions of migration, for example, toward land, are unviable) after breeding by tracking the same individuals for up to 6 years to measure route fidelity. Second, we examine potential sex-driven segregation by comparing the migration patterns of males and females. Third, to test whether dispersive migration results from intraspecific competition (or other differences in individual quality), we investigate potential relationships between activity budgets, energy expenditure, laying date, and breeding success between different routes. Daily activity budgets and energy expenditure are estimated using saltwater immersion data simultaneously recorded by the devices throughout the winter.

## METHODS

### Ethical note

All work adheres to the ASAB/ABS Guidelines for the Use of Animals in Research and was conducted after ethical approval by the British Trust for Ornithology Unconventional Methods Technical Panel (permit C/5311), Natural Resources Wales, Skomer Island Advisory Committee, and the University of Oxford. To avoid disturbance, handling was kept to a minimum, and indirect measures of variables such as laying date were preferred, where possible. Survival and breeding success of manipulated birds were monitored and compared with control birds.

### Logger deployment

Atlantic puffins are small auks (ca. 370g) breeding in dense colonies across the North Atlantic in summer and spending the rest of the year at sea. A long-lived monogamous species, they have a single egg clutch, usually in the same burrow (Harris and Wanless 2011). This study was carried out in Skomer Island, Wales, UK (51°44′N; 5°19′W), where over 9000 pairs breed each year (Perrins et al. 2008–2014).

Between 2007 and 2014, 54 adult puffins were caught at their burrow nests on a small section of the colony using leg hooks and purse nets. Birds were ringed using a BTO metal ring and a geolocator was attached to a plastic ring (models Mk13, Mk14, Mk18—British Antarctic Survey, or Mk4083—Biotrack; see Guilford et al. 2011 for detailed methods). All birds were color ringed to allow visual identification. Handling took less than 10min, and birds were released next to, or returned to, their burrow. Total deployment weight was always <0.8% of total body weight. Birds were recaptured in subsequent years to replace their geolocator. In total, 124 geolocators were deployed, and 105 complete (plus 6 partial) migration routes were collected from 39 individuals, including tracks from multiple (2–6) years from 30 birds (Supplementary Table S1). Thirty out of 111 tracks belonged to pair members.

### Route similarity

We only included data from the nonbreeding season (August–March), called “migration period” hereafter. Light data were decompressed and processed using the BASTrack software suite (British Antarctic Survey) and MatLab R2010b (MathWorks Inc.). We applied a speed filter of 500 km per day (8h of sustained flight at mean speed of 64 km/h, Pennycuick 1997) and removed the data 15 days either side of the fall and spring equinox where the latitude resolution is too low due to equivalent day length everywhere on Earth (normal resolution is ±185 km, Phillips, Silk, Croxall, et al. 2004). We calculated 2-day median positions (median latitude and longitude) for all tracks and filtered out those with high standard error (SE_longitude_ > 40 km, SE_latitude_ > 30 km). To quantify individual route fidelity, we calculated the average nearest neighbor distance (NND; in kilometer) of each migration track to all other tracks (detailed methods in Guilford et al. 2011) and compared within-individual NNDs (the variability of an individual’s route between years) with among-individual NNDs (the difference between individuals’ routes within a year). NND increases with the difference between 2 tracks, and using a 20-day temporal window allowed us to account for temporal as well as spatial route similarity—2 birds visiting the same place at different times have a larger NND than 2 birds visiting the same area within 20 days of each other. Among-individual NNDs were only calculated within years to avoid potential confounding effects of environmental conditions.

We estimated the total distance covered during each migration by summing the great-circle distances between each daily mean between August and March. Distance from the colony was calculated as the great-circle distance between the colony and each position. Distance from the colony for positions in the Mediterranean Sea was corrected to account for the flight around the Iberian Peninsula because puffins do not fly far over land.

### Activity budgets and energy expenditure

We used saltwater immersion data collected by geolocators (the proportion of time a logger spent immersed in saltwater for each 10-min interval) to estimate daily activity and energy budgets. We allocated each 10-min interval during daylight between August and March to one of 3 categories: sustained flight (≥98% dry), sitting on the water (≥98% wet), foraging (>2% dry and >2% wet, representing a succession of short flights while searching for prey and short wet bouts of sitting on the water and diving, as in Lecomte et al. 2010). At night, data were constituted of long (several hours) dry or wet bouts. Auks rarely forage or fly at night, and the dry periods observed are due to the birds tucking one leg under their wing while resting (Robertson et al. 2012; Elliott and Gaston 2014; Linnebjerg et al. 2014; Shoji et al. 2015). We therefore made the assumption that birds only rested or slept at night. Data from 4 birds carrying 2 devices for a single winter revealed that each bird tucked 1 leg preferentially (Supplementary Figure S1, see Supplementary Material for details), making estimations of nocturnal leg-tucking for birds carrying a single device highly inaccurate. Instead, we calculated the average leg-tucking time for the 4 dual-GLS birds (42% of the night) and applied this to our whole dataset to calculate sleeping and sitting time at night. We obtained a daily proportion (which therefore controls for latitudinal change in day length) of flight, foraging, and sitting behavior during day time and of resting and sitting behavior during night time. Energy costs were calculated using the daily duration spent in each activity (regardless of day length) using a model developed for guillemots *Uria aalge* (Elliott et al. 2013) and at-colony metabolic rate as a proxy for at-sea rest (Elliott and Gaston 2014). We converted our results for an average-sized 370-g puffin using the allometric equation developed for auks by Shaffer (2011) (see Supplementary Material for details).

### Phenology and breeding success

Incubation lasts ~44 days (Harris and Wanless 2011) and is shared by parents alternating shifts. Because of the difficulty of intensive direct observation in this subterranean nesting, easily disturbed species, we estimated laying date indirectly using saltwater immersion data to detect the start of incubation (see Supplementary Material for details). The accuracy of this method was verified using a subset of 5 nests that were checked daily with a burrowscope (Sextant Technology Ltd.) in 2012–2013 to determine precise laying date; its accuracy was ± 1.8 days. We calculated the birds’ postmigration laying date for 89 of the 111 tracks in our data set.

To avoid disturbance, most nests were not checked directly during the 6-week chick-rearing period following incubation, except after 2012 when a burrowscope was available. Therefore, we used a proxy for breeding success: The ability to hatch a chick and rear it for at least 15 days (mortality is highest during the first few weeks; Harris and Wanless 2011), estimated by direct observations of the parents bringing food to their chick (see Supplementary Material for details). We observed burrows at dawn or dusk when adults can frequently be seen carrying fish to their burrows for their chick. Burrows were deemed successful if parents were seen provisioning on at least 2 occasions and at least 15 days apart (this is the lower threshold used in the current method for this colony; Perrins et al. 2014). In the majority of cases, birds could be observed bringing food to their chick for longer periods. Combining the use of a burrowscope from 2012 and this method for previous years, we measured premigration and postmigration breeding success for 84 and 94 tracks in our data set, respectively.

### Sexing

For licensing reasons, we were only able to use DNA sexing in 2014, which we used to sex 20 birds using DNA extracted from feathers (Avian Biotech, UK). Birds not recaptured in 2014 were sexed behaviorally, using a conservative combination of at least 2 of 3 different measures based on morphometrics, behavioral observations at the colony, and identification of the bird taking the first incubation shift, using light and immersion data from geolocators (see Supplementary Material for details). We used the DNA-sexed birds to validate these 3 methods and obtained a 100% match with each. In total, we sexed 27 birds (13 males and 14 females), including 20 with DNA methods, which represented a total of 82 migration tracks.

### Statistics

**Figure 1:**
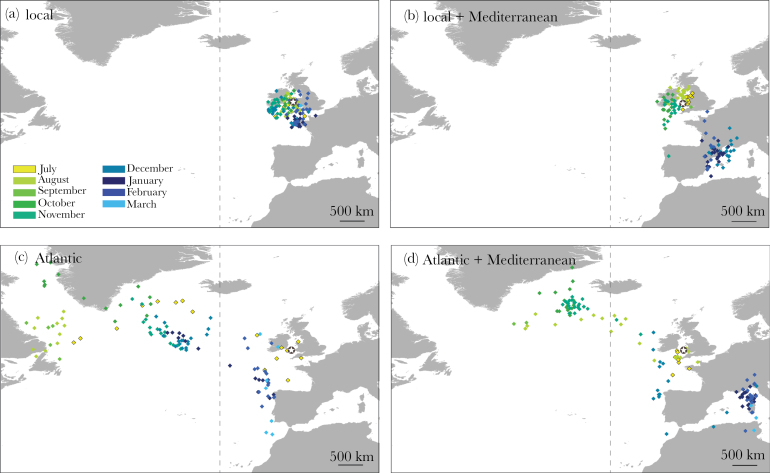
Example of each type of migration routes. Each point is a daily position. Each color represents a different month. The colony is represented with a star, the −20° meridian that was used as a threshold between “local” and “Atlantic” routes is represented with a dashed line. The breeding season (April to mid-July) is not represented. The points on land are due to low resolution of the data (~185 km) rather than actual positions on land. (a) Local (*n* = 47), (b) local + Mediterranean (*n* = 3), (c) Atlantic (*n* = 45), and (d) Atlantic + Mediterranean (*n* = 16).

We used linear and generalized linear mixed-effects models (LMMs for normally distributed data and GLMMs for Poisson and binomial distributions), always including individual and year as random effects (lmer and glmer functions, [*lme4*] package, R 3.0.2, R Core Development Team 2014). Statistical significance was obtained from comparing models to the null model (intercept + random effects). For descriptive convenience we classified routes into 4 groups using a set of quantitative criteria based on longitude thresholds, classifying separately routes going to the Mediterranean Sea, to the mid- or west-Atlantic (longitude < −20°), or to both or neither destinations (Figure 1). In all analyses of between-route differences, we excluded one of the 4 types of routes because of its small sample size (*n* = 3 vs. 16, 45, and 47 for the other types).

Differences in total distance covered, behavioral activity, and daily energy expenditure (DEE) between route types were tested with LMMs and pairwise *t*-tests. Similarity in migration phenology was tested with randomization tests (10000 iterations), for dates of arrival to and departure from the Atlantic, and departure to the Mediterranean Sea (see Supplementary Material for details). We then considered 3 potential drivers of dispersion. First, LMMs were used to investigate random dispersion (testing differences within- and among-individual route similarity). Second, we used GLMMs to test the effect of sex on migration type and distance from the colony. Spatial occupancy kernels were calculated with ArcGIS 10.0 (ESRI) and Geospatial Modelling Environment 0.7.2 (Spatial Ecology LLC) (parameters: bandwidth ~275 km, resolution ~20 km) and the overlap between sexes was calculated with the (*adehabitat*) package in R. To avoid multiple tracks from some individuals biasing the distributions, we calculated the monthly male–female overlap for each year separately, and then took the average across years. Months containing data for less than 2 males and 2 females were excluded. Finally, LMMs were used to test the effect of route type on laying date and GLMMS for breeding success (binomial distribution), and burrow was added as an supplementary random effect (because a few of the tracked birds formed breeding pairs). All means expressed in the text are ± SE. Data were log- or square root-transformed to meet parametric assumptions when necessary.

## RESULTS

### Impact

No immediate nest desertion was witnessed posthandling. Forty-five out of 54 tracked birds were recaptured in following seasons. Of the 9 birds not recaptured, all but 1 were present at the colony in at least 1 subsequent year (most were breeding but evaded recapture), giving a minimum postdeployment overwinter survival rate of 98%. The average annual survival rate of manipulated birds was 89% and their average breeding success 83%, similar to numbers obtained from control birds on the colony (see Supplementary Table S1 for details, Perrins et al. 2008–2014).

### Route diversity between birds

Individuals followed a large diversity of routes in all years, covering from 1500 to 7000 km over 8 months (Figure 1). Although some birds spent most of the winter around the British Isles, others traveled to the Northwest Atlantic, south to the Bay of Biscay and along the coast of Portugal, and to the Mediterranean Sea. The areas visited spread over 65° in longitude (from Canada to the east coast of Italy) and 36° in latitude (from the Moroccan coast to the Norwegian Sea).

Fifteen birds took a “local” route (47 tracks), 17 birds followed an “Atlantic” route (45 tracks), and 5 birds migrated to the Atlantic and then to the Med (16 tracks). Only 2 birds took a “local + Mediterranean” route (3 tracks), these were excluded from route comparison analyses to avoid likely issues with statistical power.

### Within-individual route fidelity

**Figure 2:**
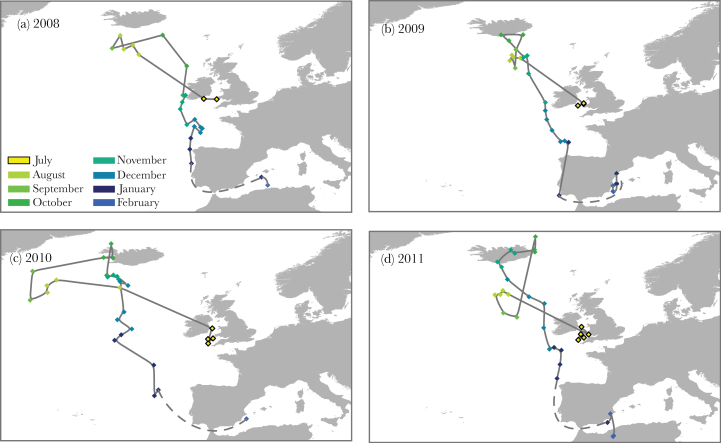
An example of spatial and temporal route fidelity during migration over 4 years. Routes shown are from a puffin tracked between 2007 and 2012. (a–d) Each position represents a 1-week median, each color represents a month. The continuous lines link the positions, the dashed lines are the probable trajectories of the bird through the Strait of Gibraltar (a straight line crossing across Spain is unlikely, as well as the crossing of Ireland). The points on land are due to low resolution of the data (~185 km) rather than actual positions on land.

Puffins showed strong individual route fidelity, with consistent migratory routes between years both spatially and temporally (Figure 2). Of 30 birds tracked for multiple (2–6) years, only 1 switched route type (Supplementary Figure S2). When comparing within- and among-individual NNDs, we found that the average NND between repeat routes of birds (358±15 km) was significantly lower than between different birds (706±12 km; LMM: *n* = 1159, ΔlogLik = 30.87, ΔAIC = −59.7, $\chi_{1}^{2}$ = 61.7, *P* < 0.001). In other words, puffin routes were more similar to their own routes in other years, than to routes from other birds that year.

### Similarity in timings within route types

**Figure 3:**
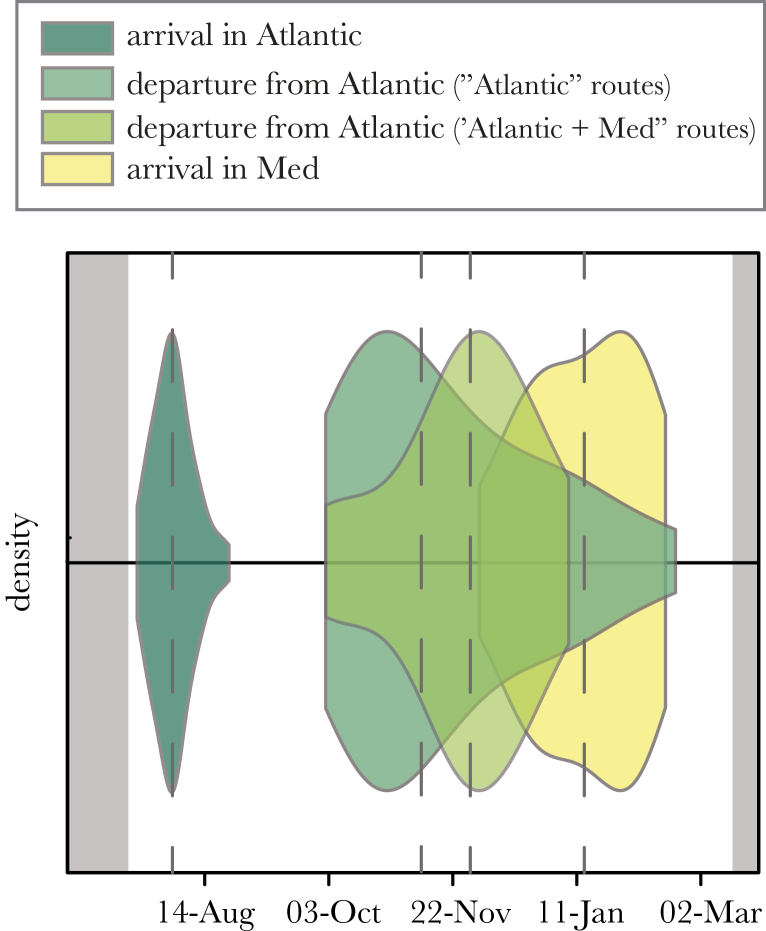
Violin plot representing the timings of migration for puffins with “Atlantic,” “Atlantic + Mediterranean,” or “local + Mediterranean” types of routes (all years pooled). The “local” routes (*n* = 47) are not represented for lack of major spatial change to describe. Each violin represents the kernel density estimation of birds (normalized) entering or leaving a specific area: entering the Atlantic (crossing the 20° meridian east to west, dark green, *n* = 61), leaving the Atlantic (crossing the 20° meridian west to east, medium green [“Atlantic” birds, *n* = 45], and light green [“Atlantic + Mediterranean” birds, *n* = 16]), or entering the Mediterranean Sea (crossing the Strait of Gibraltar west to east, yellow, *n* = 16). A narrow violin indicates that all birds depart from or arrive in an area at a similar date. The median date for each movement is indicated with a dashed gray line. The end and start of the breeding season (15 March and 15 July) are in gray.

We found similarities in the phenology of migration within route types (Figure 3). All birds migrating to the mid- or west-Atlantic crossed the −20° meridian between late July and late August (median 1 August ± 1.2 days), significantly more constrained than expected by chance (randomization test, 10000 repetitions, *P* < 0.001). Similarly, birds migrating to the Mediterranean Sea all passed the Strait of Gibraltar between late December and early February (median 13 January ± 7.3 days), a significantly smaller window than expected by chance (randomization test, 10000 repetitions, *P* < 0.001). The duration of stay in the western Atlantic was more variable: on average birds remained there 111.3±5.0 days (range: 59–200 days, median return date east: 19 November), no more constrained than expected by chance (randomization test, 10000 repetitions, *P* = 0.062). We did not calculate the duration of stay in the Mediterranean Sea because of the low resolution of the data in March due to the equinox; however, it seemed that all birds remained there until at least the end of February.

### Sex differences in migration routes

**Figure 4:**
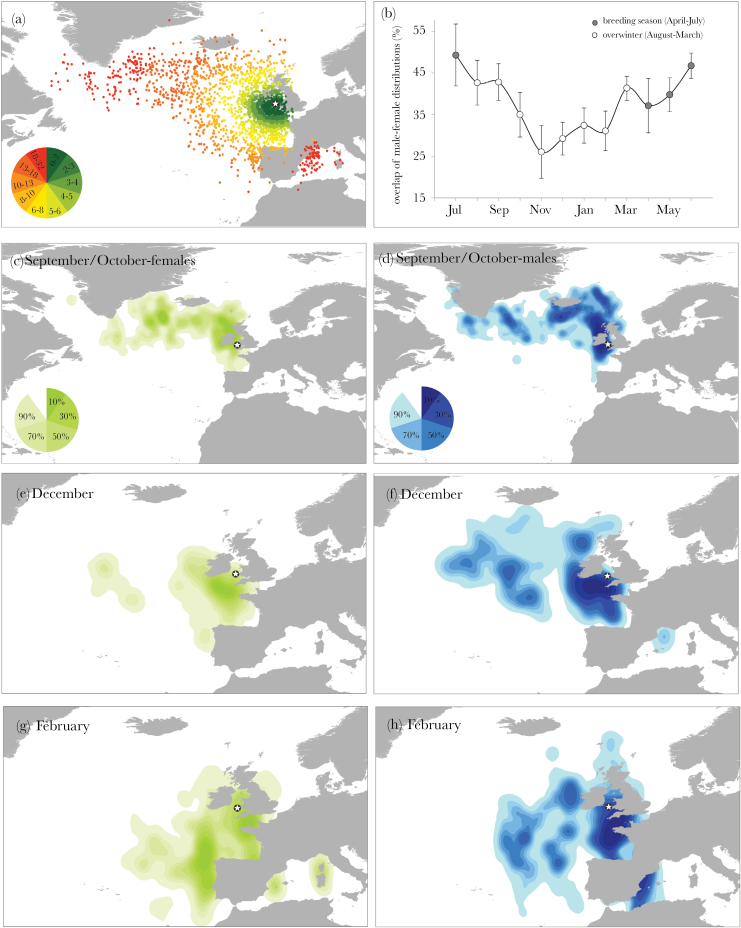
Sex differences during migration at the start, middle, and end of the migration period. (a) Distance from the colony for all our study birds, with different 10% quantiles in different colors, from green (close) to red (far). Extra-distance was added to the points in the Mediterranean Sea to account for the flight around Spain. Distances for each quantile are in the pie chart (unit: 10^2^ km). (b) Average monthly overlap (%) of the male and female 70% occupancy kernels throughout the year (mean ± SE). The overwintering months are represented with open circles and the breeding months with gray circles. (c–h) Occupancy kernels of puffins during migration for females (green, left) and males (blue, right) in September/October (c–d), December (e–f), and February (g–h). Different shades represent different levels of occupancy, from 10% (darkest) to 70% (lightest). The colony is indicated with a star.

Sex had no effect on the type of migration route, with both sexes using all types of routes almost equally (“Atlantic”: 53.8% female, “local”: 64.3% female, Atlantic_Mediterranean: 50% female; LMM: *n* = 82, ΔlogLik = 1.295, ΔAIC = 0.59, $\chi_{1}^{2}$ = 2.59, *P* = 0.940). However, after calculating distance from the colony for all birds of known sex (range: ~0–7500 km), we found a complex interaction effect between sex and month on distance from the colony (LMM: *n* = 2760, ΔlogLik = 21.2, ΔAIC = −28.5, $\chi_{7}^{2}$ = 42.51, *P* < 0.001). To investigate this interaction further, we compared the distance from the colony between sexes for each month (Figure 4a, Supplementary Figure S3). Although there were no differences between sexes at the start and end of migration, females were significantly closer to the colony in November–January. The average overlap between occupancy kernels of males and females was highest during the breeding season, but varied substantially throughout the winter (Figure 4b). It was high during the first 2 months of the nonbreeding season then decreased sharply to remain low until February, and increased again to breeding season levels in March. The distributions in (Figure 4c–h) revealed some patterns responsible for these results. From the start of migration until October the distributions were similar (Figure 4c,d). From October onwards, most females returned close to Europe, whereas many males stayed in the Atlantic, and by December only 21% of females remained in the mid-Atlantic, versus 50% of males (Figure 4e,f). From January onwards, 14% of females and 25% of males visited the Mediterranean Sea, and many individuals of both sexes stayed closer to the colony (Figure 4g,h). Although >60% of females went near the west coast of Portugal, males avoided this area and remained further from the coast in the Atlantic (33%) or elsewhere.

### Energy expenditure and activity budgets

Activity and energy budgets differed significantly among route types (Table 1a). First, the total distance covered differed significantly between the 3 main route types (LMM: *n* = 107, ΔlogLik = 15.47, ΔAIC = −26.9, $\chi_{2}^{2}$ = 30.95, *P* < 0.001, see Table 1 for pairwise comparisons). Unsurprisingly, birds staying locally covered significantly less distance than birds going to the Atlantic, which themselves covered significantly shorter distances than those going to the Atlantic and then to the Mediterranean Sea.

**Table 1:** (a) Total distance covered and DEE for each type of migration (mean ± SE and adjusted *P* values for pairwise comparison). (b) Proportions of daytime spent foraging, flying, and sitting on the surface for each type of migration route (mean ± SE and *P* values from linear mixed models with binomial family)

(a)		Distance covered (km)			DEE (kJ/day)				
Route type	*n*	Mean ± SE	Atlantic	Atlantic + Mediterranean	Mean ± SE	Atlantic	Atlantic + Mediterranean		
Local	47	4434±248	**<0.001**	**<0.001**	1049±4	0.462	**<0.001**		
Atlantic	44	5904±214	—	**<0.001**	1059±4	—	**<0.001**		
Atlantic + Mediterranean	16	7902±244	—	—	1108±9	—	—		

(b)	Foraging (% of time)			Flying (% of time)			Sitting on the water (%)		
	Mean ± SE	Atlantic	Atlantic + Mediterranean	Mean ± SE	Atlantic	Atlantic + Mediterranean	Mean ± SE	Atlantic	Atlantic + Mediterranean
Local	16.2±1.1	0.001	**<0.001**	1.9±0.4	0.231	**<0.001**	81.9±1.3	**<0.001**	**<0.001**
Atlantic	19.2±0.9	—	**<0.001**	2.5±0.4	—	**<0.001**	78.3±1.1	—	**<0.001**
Atlantic + Mediterranean	20.5±0.9	—	—	4.2±0.4	—	—	75.3±1.1	—	—

In all analyses, the “local + Mediterranean” route type is excluded because of its small sample size (*n* = 3). Significant values (*P* < 0.05) are in bold.

**Figure 5:**
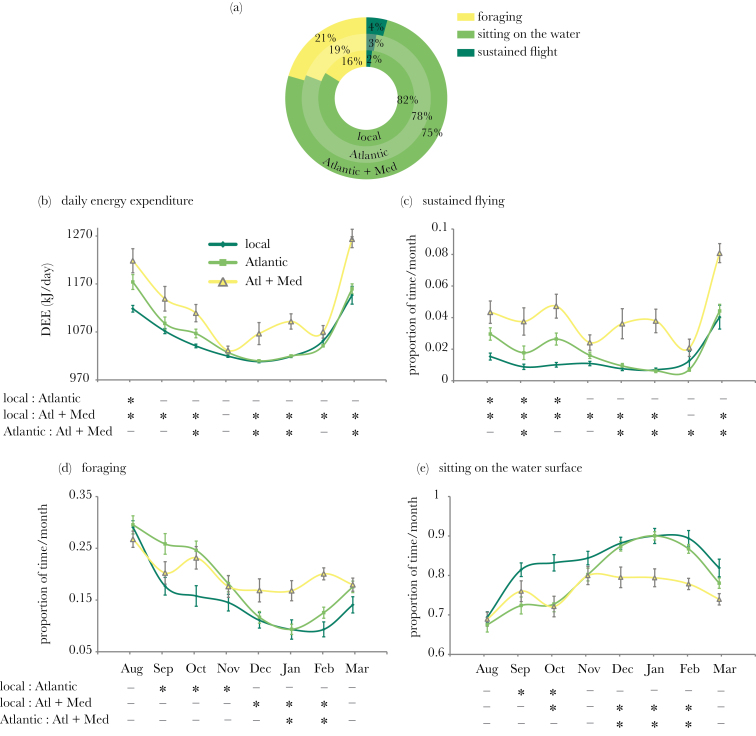
Activity budgets and average DEE for different types of routes, for the “local” (dark green), “Atlantic” (light green), and “Atlantic + Mediterranean” routes (yellow). The “local + Mediterranean” route is not included because of small sample size (*n* = 3). (a) Average winter activity budget for the 3 main routes. (b–e) Monthly average of (b) DEE and time budget of (c) sustained flight, (d) foraging, and (e) sitting on the surface for the 3 main types of routes. Means ± SE. The asterisks under the *x* axis represent significant differences (*P* < 0.05) between 2 routes (exact *P* values in Supplementary Table S2).

Second, the proportion of time spent foraging, sitting on the water, and flying, differed between route types (Figure 5a, see Table 1b for statistical tests). Birds migrating locally spent less time foraging and more time sitting on the surface than all other categories, and less time in sustained flight than birds following “Atlantic + Mediterranean” routes. On “Atlantic + Mediterranean” migrations, birds spent more time flying and foraging, and less time sitting on the water, than all others. Birds on “Atlantic” routes had intermediate levels of foraging and sitting on the surface (significantly different from the 2 other route types), but spent a similar proportion of time in sustained flight to “local” birds.

Patterns varied throughout the winter. During the first part of migration (August–November), birds in the Atlantic spent more time foraging than “local” birds, which spent more time sitting. However, during the second half of migration, “Atlantic” birds reduced their foraging dramatically to reach similar levels to “local” birds, whereas birds that left the Atlantic to go to the Mediterranean Sea continued to forage at a consistent level, also spending more time in sustained flight. The behavioral differences between birds in the Mediterranean Sea and others are not due to latitude affecting day length because behaviors are expressed as a proportion of the total daylight duration; in fact, birds in the Mediterranean Sea spent a higher proportion of a longer day foraging and flying than birds further north.

These differences in activity budgets resulted in significant differences in average DEE during the nonbreeding season (August–March) (GLMM: *n* = 94, ΔlogLik = 12.7, ΔAIC = −31.5, $\chi_{2}^{2}$ = 25.3, *P* < 0.001) (Table 1a, Figure 5b). The “Atlantic + Mediterranean” route was significantly more energy-demanding than other routes. Despite the average DEE of birds on “Atlantic” routes being higher than that of birds on “local” routes, the difference was not significant.

### Differences in breeding phenology and success between routes

To test whether birds differed in quality between routes, we compared breeding success between different types of migration routes. Breeding success did not affect subsequent migration route (GLMM: family = binomial, *n* = 78, ΔlogLik = 0.137, ΔAIC = 3.73, $\chi_{2}^{2}$ = 0.27, *P* = 0.87). However, the type of migration had a significant effect on breeding success the following season (GLMM: family = binomial, *n* = 86, ΔlogLik = 3.35, ΔAIC = −2.71, $\chi_{2}^{2}$ = 6.71, *P* = 0.035). The “Atlantic + Mediterranean” route was the most successful with 100±0% postbreeding success (*n* = 13), followed by the “local” route with 82.0±6.0% (*n* = 40); the “Atlantic” route was the least successful with 72.7±7.8% (*n* = 33). These differences could not be explained by different laying dates, as migration type did not affect subsequent laying date (LMM: *n* = 86, ΔlogLik = 0.18, ΔAIC = 3.63, $\chi_{2}^{2}$ = 0.36, *P* = 0.83). Furthermore, individual DEE, total distance covered, or the proportion of time spent foraging or flying did not explain individual differences in breeding success (GLMM: family = binomial, *n* = 76, flight: *Z* = 0.45, *P* = 0.650, foraging: *Z* = 1.10, *P* = 0.270, DEE: *Z* = −0.75, *P* = 0.45, distance covered: *Z* = −1.3, *P* = 0.19). Overall, breeding success was higher for birds that visited the Mediterranean Sea (including the 3 “local + Mediterranean” tracks) than for birds which did not (Mediterranean: 93.7±6.3% breeding success [*n* = 16], non-Mediterranean: 78.1±4.9% breeding success [*n* = 73], GLMM: family = binomial, *Z* = −218.5, *P* < 0.001).

## DISCUSSION

Atlantic puffins breeding at a major colony in the eastern north Atlantic had a strikingly dispersive migration. They visited areas across the North Atlantic and the Mediterranean Sea, often visiting several areas in the same winter. There were also large variations between routes and distances traveled. To examine the life-history significance of this variation, we used each bird’s saltwater immersion log to estimate daily activity budgets and DEE. Our estimations of DEE are in line but slightly higher than findings from studies conducted on puffins and other auks during the breeding season (Ellis and Gabrielsen 2001; Hansen 2003; Elliott et al. 2013), possibly due to migratory flights and to our classification of foraging, coarser than in Elliott et al. (2013) without diving data and directly measured metabolic rates.

“Atlantic + Mediterranean” routes were the longest and most energy consuming (with as much as a 15% increase in DEE compared with some local routes). This was reflected in a greater foraging effort (21% vs. 16% of the day on average) and less time resting on the water during the day (75% vs. 82% on average). Birds only visiting the Atlantic showed behavior consistent with these relationships, with intermediate distances, activity budgets, and energy expenditure.

These different migratory strategies were reflected in differential breeding success the following season. This is unlikely to be a simple year effect as all routes were evenly spread across years, year differences were controlled for, and average breeding success was consistent throughout the study period (Supplementary Table S1). The lack of symmetry of this effect (premigration breeding success did not differ between types of route) may be due to the lower sample size and nonsymmetrical data set (premigration and postmigration breeding success was only obtained in ~60% of tracks). Despite longer distances traveled, greater flight and foraging activity, and higher energetic costs, birds choosing to migrate to the Atlantic and then to the Mediterranean Sea had higher chances of raising a chick than birds overwintering locally or just visiting the Atlantic. This result held when including 2 other birds that visited the Mediterranean Sea after staying locally, with Mediterranean strategies leading to a significantly higher breeding success than non-Mediterranean ones. It is doubtful that dispersive migration could persist in the population if such fitness differences were sustained in the long term and there was a genetic or taught aspect to migratory routes (even indirectly, e.g., through heritability of exploratory behavior). These differences are therefore likely to be balanced by competing fitness costs and risks yet unidentified, reflect only a short window on a fitness landscape fluctuating over a longer timescale (survival and breeding success were consistently high in all but the last year), or be a response to differential quality or competitiveness among individuals.

The differences in foraging effort observed between different areas are complex and cannot be easily interpreted without data on the nature and quantity of prey caught. More foraging could equally reflect an abundance of prey, a lack of prey (birds having to forage more to catch enough prey), or birds attempting to build more reserves. The lack of correlation between foraging effort and individual breeding success suggests that it is not how much birds forage, but where they forage (and perhaps what they prey on), which affects how successful they are during the following breeding season. Interestingly, birds only visited the Mediterranean Sea, usually of low productivity, from January to March, which corresponds to the occurrence of a large phytoplankton bloom. A combination of wind conditions, winter mixing, and coastal upwelling in the north-western part increases nutrient availability (Siokou-Frangou et al. 2010), resulting in higher productivity (Lazzari et al. 2012). This could explain why these birds foraged more than birds anywhere else in the late winter and had a higher breeding success. However, we still know very little about the winter diet of adult puffins, although some evidence suggests that they are generalists (Harris et al. 2015) and that zooplankton are important (Hedd et al. 2010), and further research will be needed to understand the environmental drivers behind the choice of migratory routes and destinations.

### Potential mechanisms underlying dispersive migration

Our results shed light on 3 potential mechanisms underlying dispersive migration. Tracking individuals over multiple years (and up to a third of a puffin’s 19-year average breeding lifespan, Harris and Wanless 2011) revealed that birds consistently follow the same routes to the same approximate destinations year after year. Thus, the movements of migrating puffins did not simply result from random dispersion each year. In addition, some areas attracted many birds but others were not visited at all, suggesting that variation among individual is also not random. The individual route fidelity we observed suggests that individuals were not adapting their migrations over time—why this is the case remains to be understood. Studies of migration route fidelity in birds found that most species show at least some flexibility during their migration, with fidelity occurring only for part of the migratory journey (Dias et al. 2013; Müller et al. 2014) or in timings but not in routes (Vardanis et al. 2011; Stanley et al. 2012; Lopez-Lopez et al. 2014). Species with high consistency in routes and schedules during the entire nonbreeding season exist but seem scarcer and, so far, almost exclusively pelagic (Hunter et al. 2003; Broderick et al. 2007; Yamamoto et al. 2010; Fifield et al. 2014). Resources in the marine environment can be predictable, depending on the location and the temporal and spatial scales involved (Weimerskirch 2007). Some areas visited by our study birds are known seabird hotspots, like the area west of the mid-Atlantic ridge (Boertmann 2011; Montevecchi et al. 2012). This may lead to fidelity in stopover sites or migratory routes. Although a fixed migratory strategy may be beneficial in a predictable and stable environment but offers limited adaptability to change, individually established inflexibility can be a sign of learning-based strategies (Bonadonna et al. 2001; Paur and Gray 2011), potentially favoring flexibility over a genetically determined strategy. Some flexibility could be an adaptive advantage in the current context of rapid changes in the marine environment (Gremillet and Boulinier 2009), and the apparent high fidelity to one’s migration route over long time scales may have important implications for the species’ persistence in the future. The apparently less frequent migration route fidelity in non-marine species may reflect a more changeable environment where migrants need to respond to year-to-year changes in timings of resource availability or changing environmental conditions (e.g., Charmantier et al. 2008). It may also simply be a bias of long-term studies of individual migratory behavior toward marine species, whose longevity and breeding philopatry enable the tracking of individuals over multiple years. Although individual route fidelity allowed us to dismiss random dispersion within individuals across years, it is important to note that it could also occur among, and not within, individuals. Although we did not directly test this hypothesis, the classification of routes in 4 approximate groups and the strong similarity in the timings of major movements we observed among individuals suggest that differences among individuals are unlikely to be random.

The second mechanism we explore is spatial sex segregation, which could result from competition between sexes or differences in nutritional needs or foraging niche (Selander 1966; Ruckstuhl 2007). Although sex segregation alone is unlikely to explain the patterns we observe (it could only lead to 2 types of routes), it may be a contributing factor. Sex segregation has been observed in many sexually size-dimorphic species (Brown et al. 1995; Carbone and Owen 1995; Stewart 1997; Catry et al. 2004; Duijns et al. 2014) including seabirds (Croxall et al. 2005; Phillips et al. 2009, 2011), but examples in monomorphic species are rare (Bogdanova et al. 2011; Guilford et al. 2012; Müller et al. 2014) and the causes behind the segregation are unclear. Although we did not find any sex differences between sexually monomorphic puffins following different types of routes, we found some spatial sex segregation and sex differences in the birds’ distance from the colony. On average, the overlap between males and females was considerable during the first 2–3 months of migration but then sharply decreased, leading to substantial spatial sex segregation from November onwards. Apart from prelaying exodus in procellariiformes (Warham 1990) and occasional prebreeding trips to the mid-Atlantic in male black-legged kittiwakes *Rissa tridactyla* (Bogdanova et al. 2011), sex segregation in seabirds, and in migratory species in general, usually occurs either throughout the entire nonbreeding period (Brown et al. 1995; Stewart 1997; Marra and Holmes 2001; Phillips et al. 2011) or not at all (Guilford et al. 2009; Egevang et al. 2010; Hedd et al. 2012; Stenhouse et al. 2012). The winter diet of adult puffins is poorly known, but there seems to be no clear partitioning between sexes (Harris et al. 2015), while sexual monomorphism makes size-related segregation by dominance unlikely (Harris and Wanless 2011). To our knowledge, this is the first time that winter sex segregation of such extent is reported in auks, but the mechanisms behind such differences remain unclear and need further investigation.

Lastly, we explored the potential of intraspecific competition to drive dispersive migration. Competition for local resources leading to low-quality individuals migrating further is thought to cause differential migration in several avian species (Owen and Dix 1986; Carbone and Owen 1995; Gunnarsson et al. 2005; Bogdanova et al. 2011). Alternatively, distant productive areas in the Atlantic or the Mediterranean Sea may only be reachable by high-quality birds. Both alternatives should lead to fitness differences between routes (Alves et al. 2013). The higher breeding success of “local” birds compared with birds traveling to the Atlantic suggests a role of intraspecific competition; however, this is contradicted by birds that travel the furthest (“Atlantic + Mediterranean”) and have the highest breeding success, perhaps because they benefit from the peak in productivity in the Mediterranean Sea in late winter. If so, why only a minority of birds visit the Mediterranean Sea is puzzling. Perhaps the narrow access through the Strait of Gibraltar makes it difficult to locate or is dissuasive if puffins are adverse to land (they are not seen inshore in winter). Understanding the specific environmental conditions of these migrations and their relationship to the behavioral states is beyond the scope of this study and are unlikely to alter our findings about migratory dispersion—the key finding here is that there are different fitness consequences of different migratory routes within a single population, which has to our knowledge not been reported in a free-ranging animal.

Overall, our study provides the first in-depth insight into potential drivers and fitness consequences of dispersive migration, an unusual (but perhaps underreported) migratory pattern in animals. However, there are other potential mechanisms of dispersive migration that we could not explore here but would be interesting to investigate in future studies. Individual specialization, often related to sex, leads to spatial segregation in some seabird species (Phillips, Silk, Phalan, et al. 2004; Bearhop et al. 2006) and could potentially explain differences in migratory destinations in puffins. Testing this hypothesis would require to infer prey type from dive logger profiles (Elliott et al. 2008) or trophic level from stable isotope analysis on feather samples (Phillips et al. 2011). Age-related segregation is also commonly observed between adult and immature animals (Cristol et al. 1999; Riotte-Lambert and Weimerskirch 2013), but our study birds were all breeding adults; therefore, it is unlikely to be an important mechanism in this species. Finally, exploration–refinement (exploratory behavior during the early life followed by gradual refinement of a migration route) has also been suggested as a potential driver of dispersive migration in puffins (Guilford et al. 2011) but can only be investigated by tracking juvenile individuals over long periods, which currently remains technically challenging.

## SUPPLEMENTARY MATERIAL

Supplementary material can be found at http://www.beheco.oxfordjournals.org/

## FUNDING

A.L.F. was funded by scholarships from the Biotechnology and Biological Sciences Research Council grant ATGAAB9, Microsoft Research Cambridge, the British Council Entente Cordiale Scheme, the Mary Griffiths Foundation, and an award from the British Federation for Women Graduates. This work was funded by Microsoft Research Cambridge, the Department of Zoology of Oxford University, the RSPB, the Wilson Ornithological Society, and the Welsh Ornithological Society. We also thank Vortex Optics and donators (from #SciFund/RocketHub and the Friends of Skokholm and Skomer) for supporting this project.

## Supplementary Material

Supplementary Data
