# Supplementary material for: Drivers and fitness consequences of dispersive migration in a pelagic seabird
Source: Behav Ecol. 2016 Feb 17;27(4):1061–72. doi: 10.1093/beheco/arw013 (PMC4943109; doi:10.1093/beheco/arw013)
Supplement: Supplementary Data [file supp_27_4_1061__index.html]

Drivers and fitness consequences of dispersive migration in a pelagic seabird — Drivers and fitness consequences of dispersive migration in a pelagic seabird — Supplementary Data 

# Drivers and fitness consequences of dispersive migration in a pelagic seabird

## Supplementary Data

Data files

- Supplementary Data - Supplementary Data
- Supplementary Data - Supplementary Data
- Supplementary Data - Supplementary Data
- Supplementary Data - Supplementary Data
- Supplementary Data - Supplementary Data
