# Supplementary figures and images for: Drivers and fitness consequences of dispersive migration in a pelagic seabird
Source: Behav Ecol. 2016 Feb 17;27(4):1061–72. doi: 10.1093/beheco/arw013 (PMC4943109; doi:10.1093/beheco/arw013)

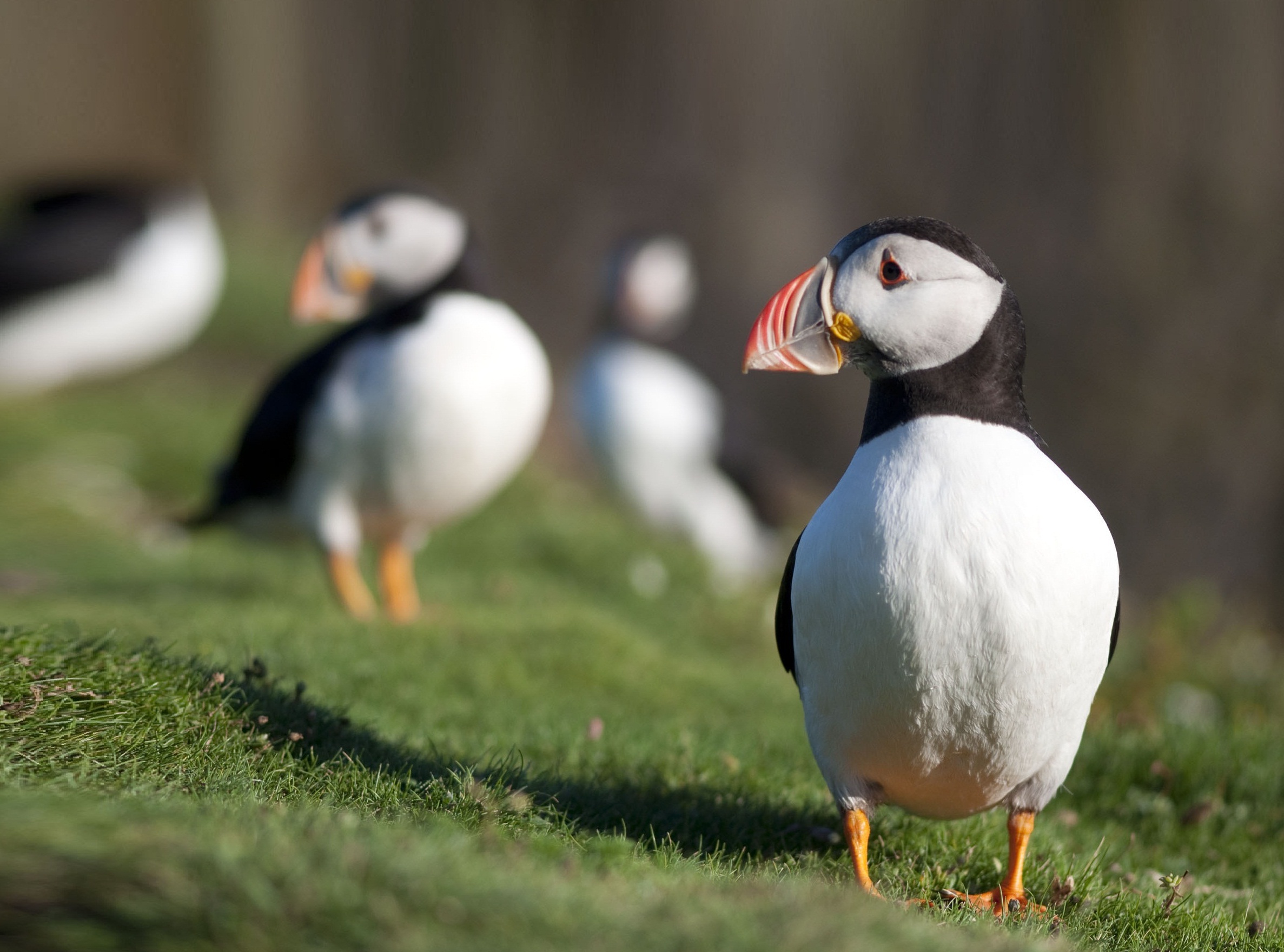

Supplement: Supplementary Data [file supp_arw013_Robin_Freeman.jpg]

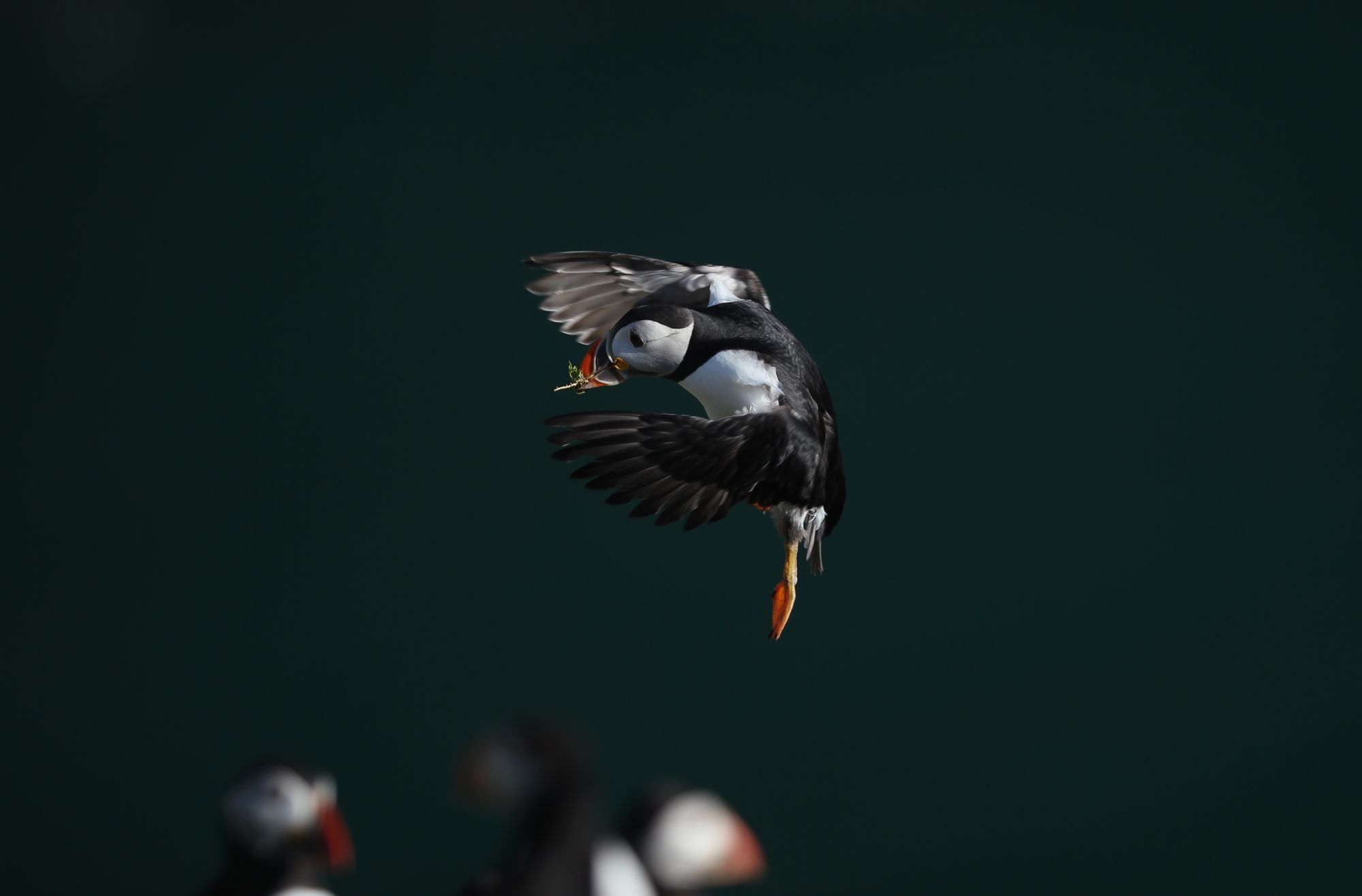

Supplement: Supplementary Data [file supp_arw013_Lewis_Yates.jpg]

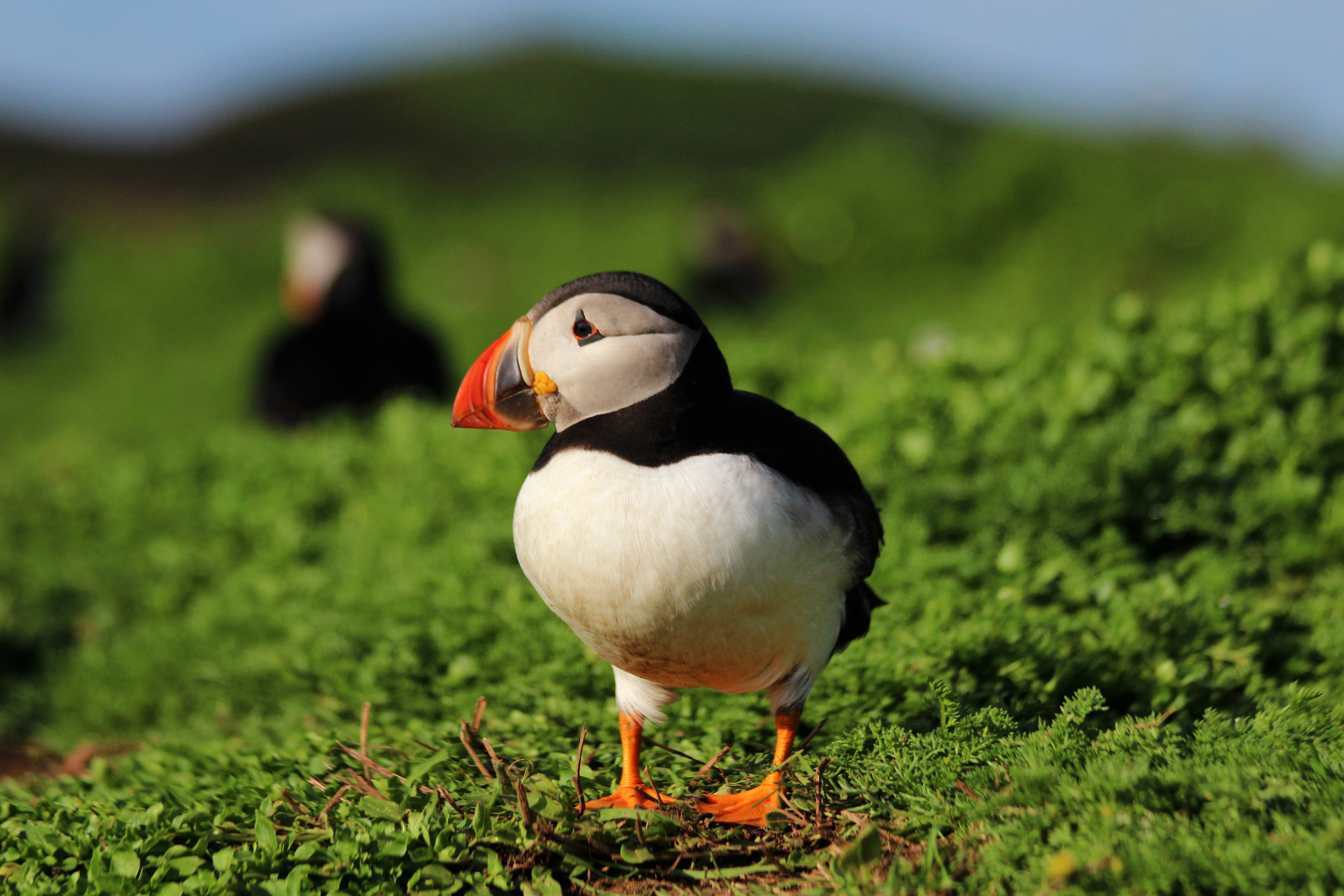

Supplement: Supplementary Data [file supp_arw013_Annette_Fayet_2.jpg]

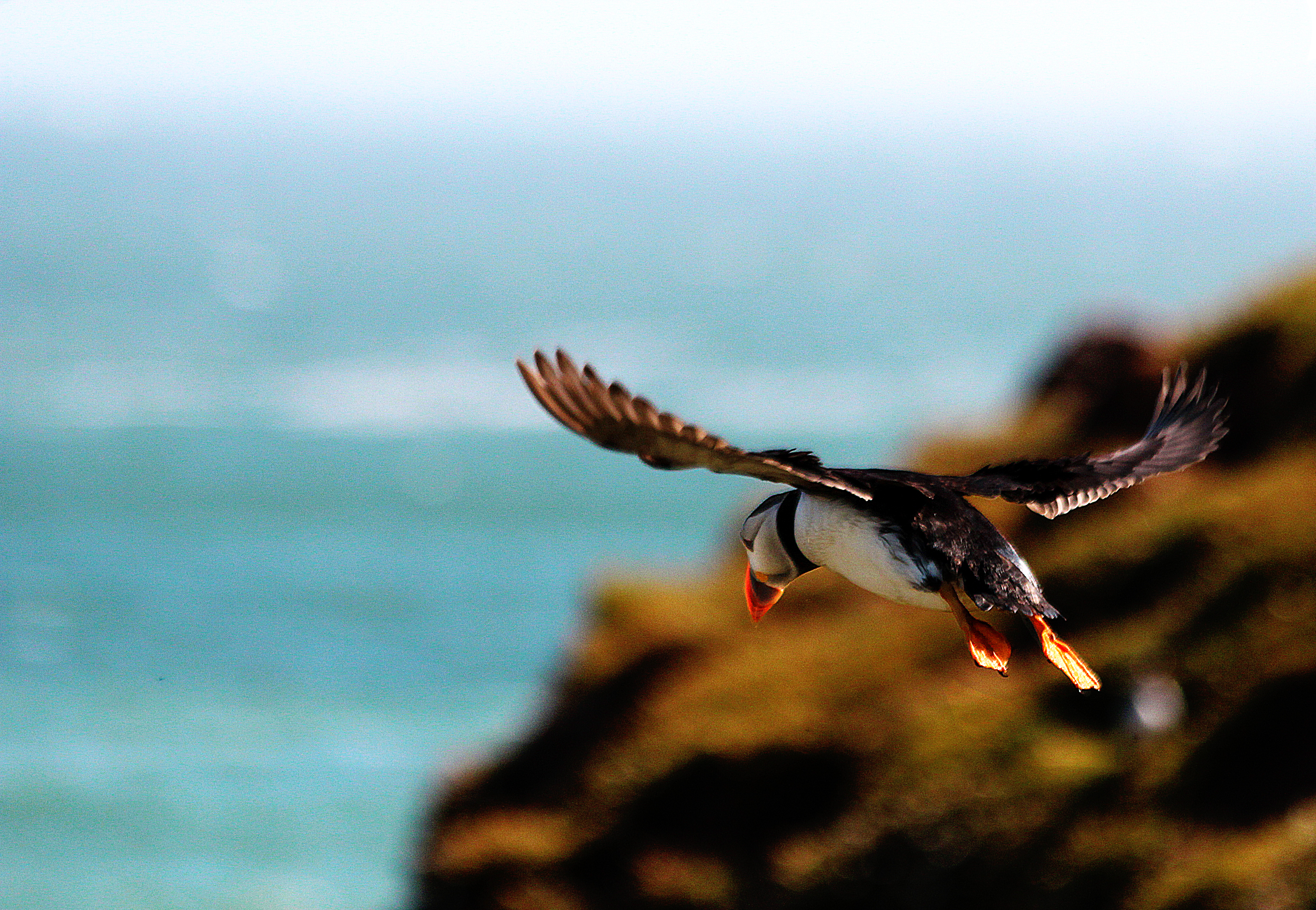

Supplement: Supplementary Data [file supp_arw013_Annette_Fayet.jpg]
