# Supplementary material for: Drivers and fitness consequences of dispersive migration in a pelagic seabird
Source: Behav Ecol. 2016 Feb 17;27(4):1061–72. doi: 10.1093/beheco/arw013 (PMC4943109; doi:10.1093/beheco/arw013)
Supplement: Supplementary Data [file supp_arw013_Supplementary_Material_BE_final.pdf]

SUPPLEMENTARY MATERIAL

Supplementary Methods

Estimating leg-tucking at night

We estimated leg-tucking by using activity and light data for each 10min interval. We assumed that there was no foraging or flight at night and that the dry periods observed were due to the birds tucking one leg under their wing while sleeping (Robertson et al., 2012; Linnebjerg et al., 2014). However, using data from 8 geolocators deployed simultaneously on the 2 legs of 4 birds during the 2010-2011 winter, we found that each bird was tucking one leg preferentially to the other (Fig S01). Therefore, instead of multiplying the proportion of nightly dry time by two we calculated the average leg-tucking (i.e. sleeping) time for these four birds (42% of the night) and applied this to our dataset to calculate time sleeping and resting at night. All four birds spent very little time with both legs up (~0.15% of the night), presumably tucking both under their wings.

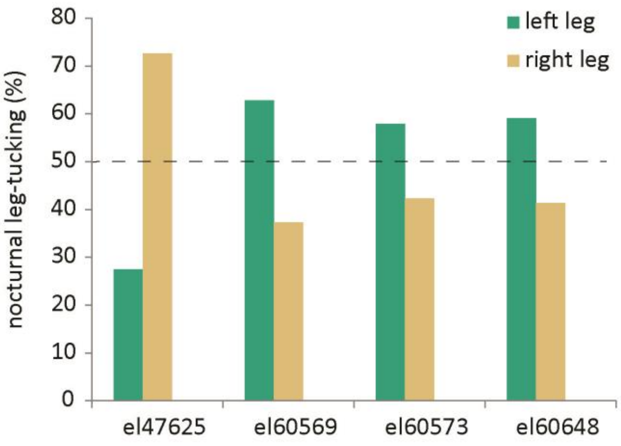

Figure S01. Proportions of left and right leg tucking at night by 4 puffins during the 2010-2011 winter.

Energy costs

We used a model developed on murres from (Elliott et al., 2013) to calculate energy costs:

$$DEE\ (Watts) = 8.9 * T_R + 148 * T_F + 28 * T_{WS} + 27 * T_D$$

where in the original equation T_R is the resting time at the colony, T_F time in flight, T_{WS} time sitting on the water surface and T_D diving time. Here we use time foraging as a proxy for diving time and time sleeping (tucked leg) as a proxy for resting time (there is evidence that resting time at the colony is similar to resting on the surface (Elliott & Gaston, 2014)). We then converted our results to kJ/day and use the allometric equation developed for auks in (S. A. Shaffer, 2011) to adjust the results to a 370g puffin.

$$Field\ Metabolic\ Rate\ (kJ.day^{-1}) = 15.537 * Mass^{0.689}$$

Laying date

Incubation generally lasts around 44 days (Harris and Wanless 2011) and is shared by parents alternating shifts while the other forages. Because of the difficulty of intensive direct observation in this subterranean nesting, easily disturbed species, we estimated laying date indirectly using salt-water immersion data to detect the start of incubation. For those pairs in which we tracked both birds, lay date was identified as the start of the first continuous 6 h daytime period in which one or other bird's immersion logger was dry (i.e. combined activity showed consistently that at least one parent was not on the water). This method also allowed us to detect failure of the first egg followed by relaying (Figure S1). When only one bird of a pair was tracked, we estimated lay date as the start of the bird's first incubation shift. The accuracy of this method was verified using a subset of 5 nests which were checked daily with a burrowscope (Sextant Technology Ltd.) in 2012-2013 to determine

precise laying date; its accuracy was ± 1.8 days. We calculated the birds' post-migration laying date for 89 of the 111 tracks in our dataset.

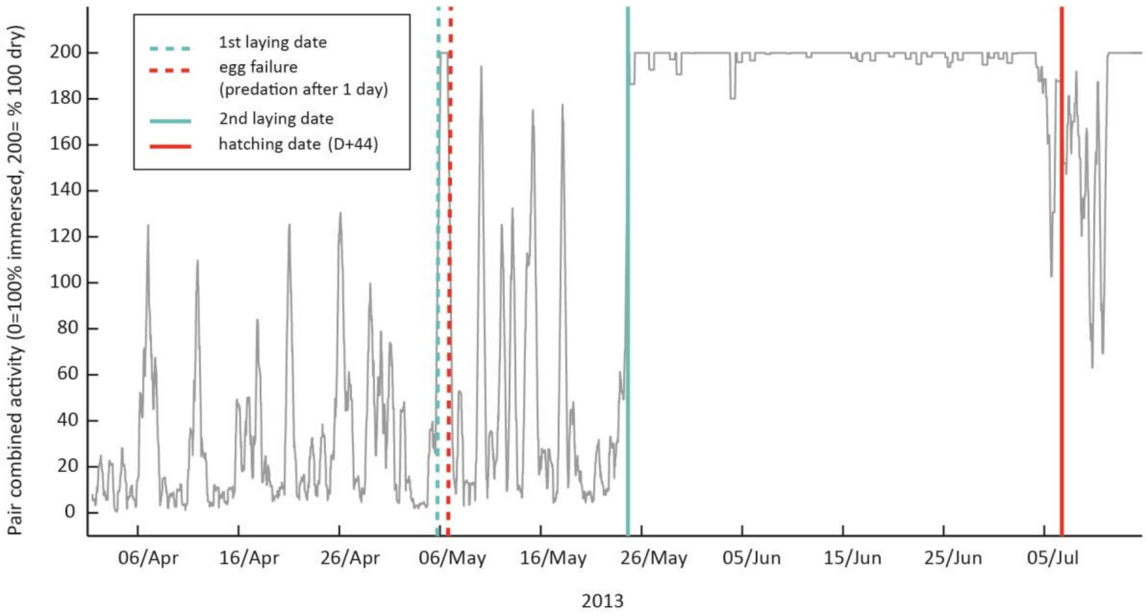

Figure S1 – Combined immersion activity for an example pair during the pre-laying and incubation period of 2013. Lay dates (defined as the start of a long (>6h) dry period) are in blue. The first egg was lost to predation the day after it was laid (pers. observation), the 2nd egg successfully hatched. The end of incubation is indicated with a red line: in the first case after predation of the first egg, the second (hatching) 44 days after relaying (in this case presence of the chick was verified with a burrowscope). During these 44 days there was always an adult present on the colony, except for short bouts of maximum 1min.

Breeding success

We did not check nests directly during the 6-week chick-rearing period following incubation to minimise disturbance, except after 2012 when a burrowscope was available. Nests which failed during incubation (unsuccessful incubation, egg damaged or infertile) were identified using the same immersion data technique used to identify laying date: nests with an incubation period unusually short or long, i.e. outside of a 14-day window around the average 47 days estimated from our dataset, were deemed unsuccessful.

For the nests which manage to hatch a chick, we used a proxy for breeding success: the ability to hatch and rear a chick for at least 15 days (mortality is highest during the first few weeks; (Harris and Wanless 2011)). This was estimated by direct observations of the parents breeding beakfuls of sandeels to their burrow. During the chick-rearing season (late May to early July) we regularly observed burrows at dawn or dusk when feeding is most likely to occur (observation took place for a few hours several times a week during this whole period, each year). The birds were deemed to have a successful season if they were seen provisioning their chick at least 15 days apart (i.e. their chick reached at least 15 days, this is the lower threshold used in the standard method for this colony: Skomer Island seabird monitoring reports 2008-2014). Although 15 days was our cut-off value, in the most cases birds could be observed bring food to their chick for longer periods.

Sexing

We sexed 20 birds using DNA extracted from 5 breast feathers plucked during device retrieval (Avian Biotech, UK). 7 additional birds which could not be recaptured in 2014 were sexed behaviourally, using a conservative combination of at least 2 of 3 different measures. First, we compared bill size between partners, the main method used to sex puffins before DNA methods; females usually have a smaller beak than their partner (Corkhill 1972; Friars and Diamond 2011). Second we used repeated observations (conducted for 1-2h at least 1-2 times a week during the 2007-2013 breeding seasons) of typical male-behaviour at the colony (e.g. head-flicking display (Taylor 2011) and propensity to fight (Creelman and Storey 1991)) or copulation. Finally, when both members of a pair were tracked with a geolocator, we used the technique used to measure laying date to identify the bird which took the first incubation, which tends to be male in many other burrow-nesting seabirds (Warham 1990; Pinet et al. 2012). We used the DNA-sexed birds to validate these 3 methods. The accuracy obtained were 100% for the bill-size method (validated on 7 pairs), 100% for behavioural observations (validated on 10 pairs), and 100% for the first incubation shift method (validated in 6 pairs). However, to be conservative we only included the 7 birds for which we had results from 2 or 3

methods in our analysis of sex-differences (7 other birds for which we only had one of the 3 measurements were excluded). In one pair (which we did not include in our analysis), one of the three methods was in disagreement with the two others, which shows that none of these methods apart from DNA sexing are 100% reliable (despite our 100% validation), which is we only included birds for which we had 2 or 3 (agreeing) measures. In total we sexed 27 birds (13 males and 14 females), including 20 with DNA methods and 7 with behavioural methods.

Migration phenology – randomisation tests

We used randomisation tests to test whether the timings of major movements were more similar between birds than expected by chance. We defined major movements as entering and leaving the mid-west Atlantic (crossing the -20° meridian), and entering the Mediterranean Sea (crossing the Strait of Gibraltar). Because of the proximity to the spring equinox we could not collect data on leaving the Mediterranean Sea. For each bird the date of interest was extracted (e.g. entering the Atlantic) as well as the duration of stay in the Atlantic or in the Med (assumed in this case to be until the 28th February). A date was randomly sampled from any possible date during the 31st July – 28th Feb window which would allow the bird to remain in the Atlantic or Mediterranean Sea for the same duration as its actual stay. For example, if a bird stayed for 100 days in the Atlantic, a date as randomly sampled from the 31st July-20th November window (20th November = 28th Feb - 100 days). We restricted the full window of dates to the 31st July – 28th Feb, which corresponds to ~ 2 weeks after the end of breeding and before return to the colony, to allow for leaving/returning to the colony. This was done for all birds visiting the Atlantic and/or the Mediterranean Sea and the range of dates obtained was compared to the actual range. The process was repeated 10,000 times and a P-value calculated. If $P < 0.05$, the event was considered significantly more constrained than expected by chance.

References

Corkhill, P. (1972). Measurements of Puffins as Criteria of Sex and Age. *Bird Study*, *19*(4), 193–201.

Creelman, E., & Storey, A. E. (1991). Sex Differences in Reproductive Behavior of Atlantic Puffins. *The Condor*, *93*(2), 390–398.

Elliott, K. H., & Gaston, A. J. (2014). Dive behaviour and daily energy expenditure in Thick-billed Murres *Uria lomvia* after leaving the breeding colony. *Marine Ornithology*, *42*(2), 183–189.

Elliott, K. H., Ricklefs, R. E., Gaston, A. J., Hatch, S. A., Speakman, J. R., & Davoren, G. K. (2013). High flight costs, but low dive costs, in auks support the biomechanical hypothesis for flightlessness in penguins. *Proceedings of the National Academy of Sciences*, *110*(23), 9380–9384.

Friars, K. A., & Diamond, A. W. (2011). Predicting the Sex of Atlantic Puffins, *Fratercula arctica*, by Discriminant Analysis. *Waterbirds*, *34*(3), 304–311.

Harris, M. P., & Wanless, S. (2011). *The Puffin*. London: T. & A.D. Poyser.

Linnebjerg, J., Huffeldt, N., Falk, K., Merkel, F., Mosbech, A., & Frederiksen, M. (2014). Inferring seabird activity budgets from leg-mounted time-depth recorders. *Journal Für Ornithologie*, *155*(1), 301–306.

Perrins C, Boyle D, Baer J, Bueche B, Cole T, Kipling R, Milborrow J, Stubbings E, Taylor C, Yates L. Seabird monitoring on Skomer Island 2008-2014.

Pinet, P., Jaquemet, S., Phillips, R. A., & Le Corre, M. (2012). Sex-specific foraging strategies throughout the breeding season in a tropical, sexually monomorphic small petrel. *Animal Behaviour*, *83*(4), 979–989.

Robertson, G. J., Fifield, D. A., Montevecchi, W. A., Gaston, A. J., Burke, C. M., Byrne, R., … Wilhelm, S. I. (2012). Miniaturized data loggers and computer programming imrove seabird risk and danger assessments for marine oil spills in Atlantic Canada. *Journal of Ocean Technology*, *7*, 42–58.

Shaffer, S. A. (2011). A review of seabird energetics using the doubly labeled water method. *Comparative Biochemistry and Physiology a-Molecular & Integrative Physiology*, *158*(3), 315–322.

Taylor, K. (2011). Puffin behaviour. In *The Puffin*. London: T. & A.D. Poyser.

Warham, J. (1990). *The Petrels: Their Ecology and Breeding Systems*. London, UK: Academic Press.

Supplementary Figures (S2-S3)

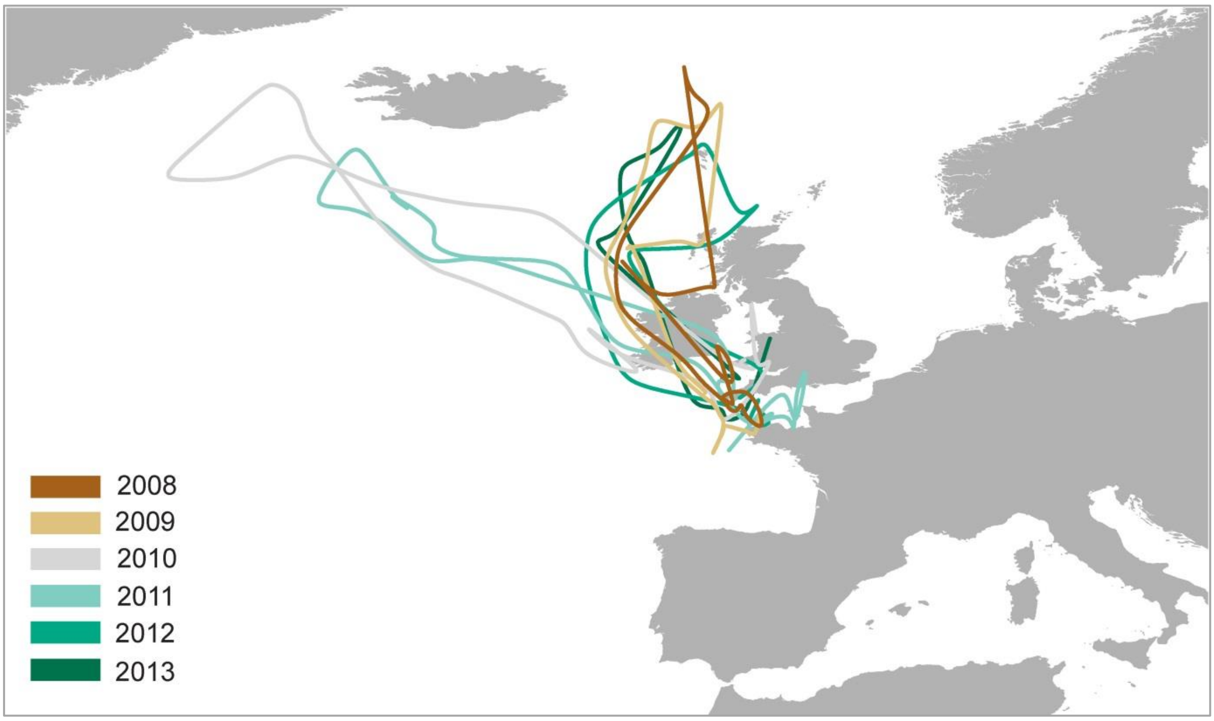

Figure S2. Switch of migratory route over 6 years in a puffin. Data obtained between 2008 and 2014 on puffin EJ47626. The trajectories are straight lines between weekly medians (July-March), smoothed over 3 decimal degrees. Each colour represents one year (see legend). This bird had a "local" migration route in 2008, and 2009, switches to an "Atlantic" route in 2010 and 2011, and reverses back to "local" in 2012 and 2013. It is the only bird in our dataset which shows such a clear switch in migration route.

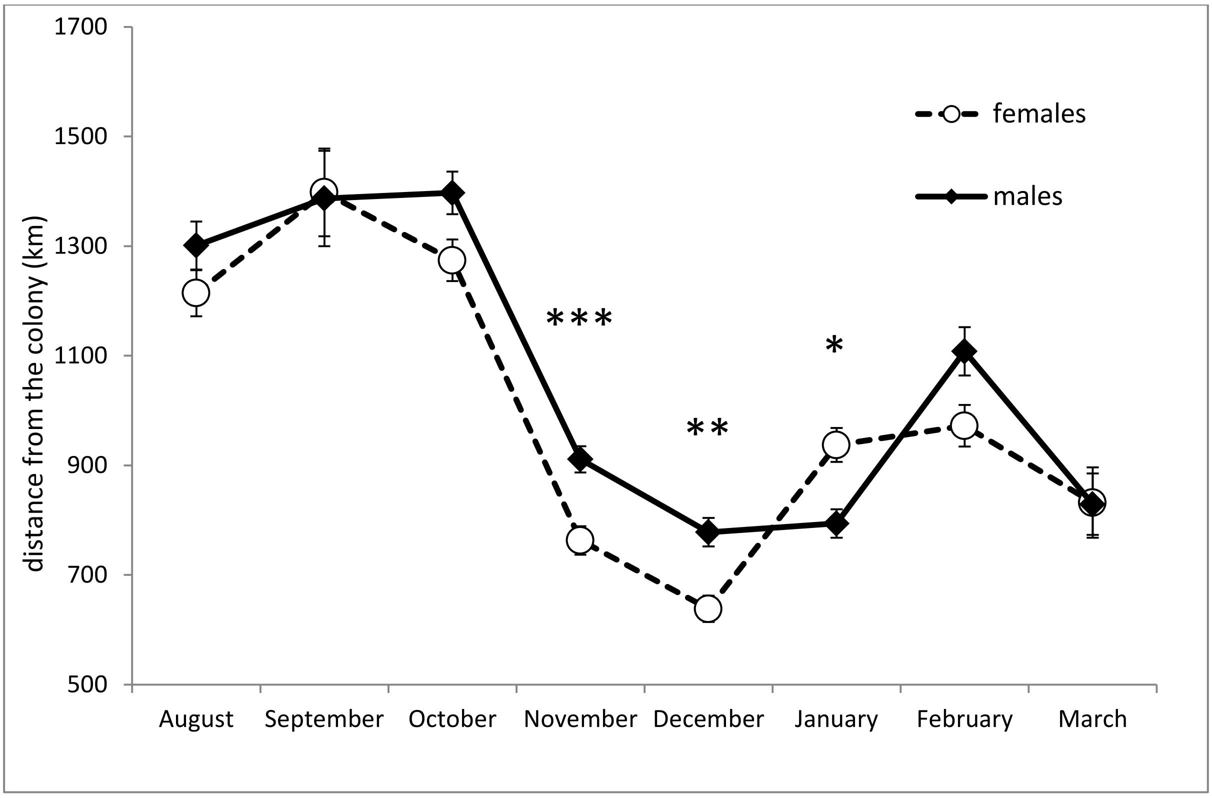

Figure S3. Average monthly distance from the colony for both sexes. The males are represented with black diamonds and a continuous line, the females with open circles and a dashed line. Data presented are mean ± SE. Asterisks indicate significant differences between males and females (Wilcoxon tests, *: $P < 0.05$; **: $P < 0.01$; *** : $P < 0.001$).

Supplementary Tables (S1 – S2)

Puffin	2007	2008	2009	2010	2011	2012	2013	2014
EB80612	/	/	/	/	/	D	R	wreck *
EB80796	/	/	/	/	/	D	R	
EB80848	/	/	/	/	/	D	R	R
EJ09593	/	D	R	R	R - 2		R	
EJ47617	/	D	R	R	R	colony	R	colony
EJ47621	/	D			colony			
EJ47622	/	D		R	colony	R		
EJ47623	/	D	R		R		R	wreck *
EJ47624	/	D	R		colony	R	colony	R
EJ47625	/	D	R	R	R	R	R	
EJ47626	/	D	R	R	colony	colony	R -11	wreck *
EJ99351	D	R	R	R				
EJ99352	D	R			R			
EJ99354	D	removed	/	/	/	/	/	/
EJ99355	D	colony	R	R	R	R	R	colony
EJ99411	/	D	R	R	R	colony	colony	colony
EJ99416	/	D						
EJ99417	/	D	R	R		R	3	
EJ99419	/	/	D					
EJ99420	/	D						
EJ99424	/	D		R	colony	colony		R
EJ99427	/	D	R	R	R	colony	R	
EK59012	/	/	/	/	/	D	R	
EL60569	/	/	D	R	R		R	colony
EL60571	/	/	D	R		R		
EL60572	/	/	D	R	colony			
EL60573	/	/	D	R	R	R	R - 13	
EL60575	/	/	D	R				
EL60579	/	/	D	R	R	R	colony	
EL60647	/	/	/	/	/	D		
EL60648	/	/	D	R	R		R -7	
EL60862	/	/	/	D	colony	R		R
EL60863	/	/	/	D	R	R	R	R
EL60866	/	/	/	D	colony	colony		
ET43490	D	R	R	R	R	R	R	R
EX83106	/	/	/	/	D	colony		
EX83181	/	/	/	/	D - 2		R	R

EX93522	/	/	/	/	/	D		colony
EX93804	/	/	/	/	/	/	D	wreck *
EY07414	/	/	/	/	/	D	colony	
EY07420	/	/	/	/	/	D		
EY07421	/	/	/	/	/	D	R	R
EY07422	/	/	/	/	/	D		colony
EY07423	/	/	/	/	/	D	R	colony
EY07424	/	/	/	/	/	D	R	colony
EY07425	/	/	/	/	/	D	R	
EY07259	/	/	/	/	/	D	R	wreck *
EY07260	/	/	/	/	/	D	R	
EY07261	/	/	/	/	/	D	R	
EY07262	/	/	/	/	/	D	R	R
EY07263	/	/	/	/	/	D	R	
EY07264	/	/	/	/	/	D	R	R
EY07265	/	/	/	/	/	D	R	
EY07267	/	/	/	/	/	D	R	R
	**2007**	**2008**	**2009**	**2010**	**2011**	**2012**	**2013**	**2014**
GLS birds survival rate	n/a	100 % (5/5)	100% (18/18)	92.3% (24/26)	92.6% (25/27)	92.3% (24/26)	91.1 % (41/45)	52.4 % (22/42) *
colony survival rate	84.70%	93.90%	93.40%	83.80%	93.60%	97.80%	84.2 %	59.5 % *
GLS birds breeding success	/	87.5% (14/16)	80% (20/25)	84.6% (22/26)	100% (11/11)	74.1% (20/27)	83.9 % (26/31)	71.4 % (10/14)
colony breeding success	79%	63%	77%	80%	84%	80%	78%	53 %

Table S1. Details of geolocator deployments on puffins since 2007. The table indicates when the birds were captured for a first geolocator deployment (D), recaptured (R), if their device was removed and not replaced ("removed"), or if the birds were present at the colony but not recaptured ("colony"). The background colour indicates breeding success: green indicates that the birds successfully hatched a chick which survived over 2 weeks (i.e. the parents were seen bringing fish to their burrow at least two weeks after hatching date), yellow indicates that a chick hatched but it is unknown whether it survived for over two weeks. Orange indicates that the birds failed to lay or to hatch an egg. White indicates an unknown reproductive success (but the birds were seen at the colony). Grey indicates that the birds were not seen at all at the colony. The number next to some letters indicate the minimum age of the chick when the parents were last observed feeding it (above 14 is here considered as successful breeding success). The survival rate and breeding success of study birds can be found

for each year at the bottom of the table, along with the annual general survival rate and breeding success of the colony as a whole (Skomer Island Seabird Reports 2008-2014).

* In February 2014, exceptionally violent storms left 28,000 puffins wrecked on the French Atlantic coast, 5 (~10%) of our study birds were found dead among them (marked “wreck” in the table). Approximately half of our study birds were missing from the colony in summer 2014 – a number much higher than usual but equivalent to the numbers obtained on a colour-ring study on a different part of the colony.

	Aug	Sep	Oct	Nov	Dec	Jan	Feb	Mar
**Flight**								
local_Atlantic	**0.004**	**0.010**	**0.001**	0.103	0.406	0.693	0.063	0.946
local_Atlantic + Med	**0.000**	**0.000**	**0.000**	**0.023**	**0.000**	**0.000**	0.190	**0.018**
Atlantic_Atlantic + Med	0.166	**0.048**	0.054	0.287	**0.000**	**0.000**	**0.005**	**0.026**
**Forage**								
local_Atlantic	0.976	**0.005**	**0.003**	0.299	0.754	0.983	0.059	0.176
local_Atlantic + Med	0.976	0.461	0.076	0.607	0.079	**0.024**	**0.000**	0.239
Atlantic_Atlantic + Med	0.976	0.215	0.678	0.854	0.088	**0.024**	**0.003**	0.863
**Sitting on water surface**								
local_Atlantic	1.000	**0.004**	**0.001**	0.215	0.700	0.989	0.209	0.226
local_Atlantic + Med	1.000	0.289	**0.009**	0.321	**0.006**	**0.001**	**0.000**	0.054
Atlantic_Atlantic + Med	1.000	0.326	0.867	0.935	**0.009**	**0.001**	**0.005**	0.226
**Daily Energy Expenditure**								
local_Atlantic	**0.015**	0.280	0.154	0.287	0.866	0.814	0.113	0.338
local_Atlantic + Med	**0.002**	**0.008**	**0.001**	0.377	**0.001**	**0.000**	0.805	**0.009**
Atlantic_Atlantic + Med	0.164	0.058	**0.015**	0.910	**0.001**	**0.000**	0.162	**0.001**

Table S2. Test results investigating differences between route types in proportions of time spent flying, foraging and sitting, and daily energy expenditure, for each month of the migration period, as presented on Figure 2. The *P* values are obtained from GLMMs and pairwise comparisons with adjustment for multiple comparisons. Significant p-values (<0.05) are in bold.
